# The body politic: the relationship between stigma and obesity-associated disease

**DOI:** 10.1186/1471-2458-8-128

**Published:** 2008-04-21

**Authors:** Peter Muennig

**Affiliations:** 1 Department of Health Policy and Management, Mailman School of Public Health, Columbia University, New York, USA

## Abstract

**Background:**

It is commonly believed that the pathophysiology of obesity arises from adiposity. In this paper, I forward a complementary explanation; this pathophysiology arises not from adiposity alone, but also from the psychological stress induced by the social stigma associated with being obese.

**Methods:**

In this study, I pursue novel lines of evidence to explore the possibility that obesity-associated stigma produces obesity-associated medical conditions. I also entertain alternative hypotheses that might explain the observed relationships.

**Results:**

I forward four lines of evidence supporting the hypothesis that psychological stress plays a role in the adiposity-health association. First, body mass index (BMI) is a strong predictor of serological biomarkers of stress. Second, obesity and stress are linked to the same diseases. Third, body norms appear to be strong determinants of morbidity and mortality among obese persons; obese whites and women – the two groups most affected by weight-related stigma in surveys – disproportionately suffer from excess mortality. Finally, statistical models suggest that the desire to lose weight is an important driver of weight-related morbidity when BMI is held constant.

**Conclusion:**

Obese persons experience a high degree of stress, and this stress plausibly explains a portion of the BMI-health association. Thus, the obesity epidemic may, in part, be driven by social constructs surrounding body image norms.

## Background

Over the past two decades, body mass has increased rapidly, especially among persons who are already overweight or obese.[1] This increase in body mass presents public health challenges both because thin bodies are seen as physically attractive, and because obesity is associated with poor health outcomes.[2,3] Obese persons' health is worse throughout the lifecourse than normal weight persons, and their lifespan is nearly two years shorter on average.[2]

The conventional wisdom is that this obesity-related excess mortality arises from a series of biochemical changes associated with adiposity. [4-14] These changes include higher serum levels of pro-inflammatory, pro-thrombotic cytokines, such as C-reactive protein (CRP), tumor necrosis factor alpha (TNF-alpha), and interleukin-6 (IL-6).[15] Cytokines serve as messenger molecules that mobilize the immune system, increase vascular tone, and enhance the formation of blood clots. Most of these latter functions offer a huge survival benefit when an organism is threatened with injury. However, cytokines can also have harmful effects when serum levels remain chronically elevated, including an increased risk of diabetes, hypertension, heart disease, and immune dysfunction.[16]

The idea that adipose cells either secrete these biochemical mediators or induce other cells to secrete them has been called the adiposity hypothesis.[10,13,17] In addition to elevated cytokine levels, a wider array of autonomic and hormonal dysfunction has been observed in obese persons, and this dysfunction also plausibly contributes to obesity-associated morbidity and mortality.[17]

Nevertheless, some scientists suggest that researchers have incited a "moral panic" over the obesity epidemic without forwarding sufficient proof that obesity causes poor health.[18,19] They argue that there is certainly a strong correlation between adiposity and poor health, but that this correlation is not causal. They point to phenomena that cannot be readily explained by the adiposity hypothesis. For instance, overweight people who are not obese suffer less morbidity and mortality than normal weight people.[3] Moreover, some groups, such as obese African Americans, do not suffer from excess mortality the same way that obese whites do.[20,21] More troubling, there is no direct evidence that expanded adipose cells themselves actually secrete sufficient quantities of biochemical mediators to explain the additional burden of disease suffered by obese persons.[13]

There is also a complementary explanation for the association between obesity and the presence of higher serological levels of pro-inflammatory, pro-thrombotic cytokines; the stigma associated with obesity could explain the higher serum levels of these very mediators. [22-24] Psychological stress causes general autonomic activation. When chronic, as in the case of stigma, post-traumatic stress disorder (PTSD), or low socioeconomic status, stress may disrupt the homeostatic mechanisms that check the levels of the same cytokines that have been observed to be elevated in obese persons.[16,17,25,26] This disruption of homeostatic systems has been hypothesized to lead to a new set point for these mechanisms in a phenomenon called allostatic load. In most chronically stressed individuals, this set point is higher, leading to elevated levels of these biochemical stress mediators. [26-29] Allostatic load may explain higher rates of these mediators seen in a wide array of groups experiencing psychological stress, such as African-Americans who experience racial discrimination on a daily basis.[30]

Likewise, obese persons report extremely high levels of stigmatization and discrimination; among one group of formerly obese persons asked to choose between blindness or obesity, 89% chose blindness.[31] Discrimination is also pervasive; there is evidence that parents discriminate against their obese children, doctors against their obese patients, and husbands against their obese wives.[32,33] All things otherwise equal in childhood, heavy people are less likely to reach milestones of social success, including completing schooling, finding a romantic partner, and garnering a good job.[34] As a result, obese persons are more likely to suffer from a negative self-image than thinner persons.[18,33,34]

In this paper, I explore the possibility that the stress associated with social stigma and negative body image among obese persons explains some of the weight-associated morbidity that researchers had previous attributed to adiposity alone. I will critically re-examine the scientific literature and draw upon my own research to explore interlocking lines of evidence supporting the proposed obesity-related stigma hypothesis. After a brief review of the conceptual framework of the paper, I will go into each of the lines of evidence supporting my hypothesis in detail. I will conclude with a brief discussion of the policy implications of this hypothesis and the limitations of the analytical framework I present.

## Conceptual framework

To my knowledge, this paper forwards the first examination of stigma-induced psychological stress as a putative etiologic agent in the pathophysiology of obesity. In assembling this paper, it was therefore necessary to think through various ways in which psychological stress might be shown as a contributor to morbidity and mortality. To do so, I examined four lines of evidence suggesting that stigma-induced stress mediates the relationship between obesity and health.

First, I re-examine studies embedded in the adiposity literature as well as studies from the literature on socioeconomic status-induced stress and health (in which BMI was included as a covariate). While metabolic studies of obesity simply measure serum levels of cytokines and other compounds, studies examining socioeconomic status-induced stress tend to stress subjects in a laboratory setting. In doing so, they provide a more direct measure of how being obese or thin modifies the normal human stress response.[10,11,28] The socioeconomic status literature also contributes by using much larger, nationally representative samples.[35,36] Finally, body mass index plays a prominent role in some of the more groundbreaking stress studies, including those linking stress to markers of premature biological aging.[37] However, it is difficult to ascertain causality from these simple associations; BMI may be inducing the stress response, adipose tissue may be producing the stress mediators, BMI may be a confounder, or stress may lead to an increase in BMI.[16]

Second, to better tease these factors apart, I and a team of researchers sought to ascertain whether it was one's BMI or one's satisfaction with his or her weight that was most important in the relationship between adiposity and health.[38] To do this, we used the 2003 Behavioral Risk Factor Surveillance System dataset, which contains a question asking subjects to place a numerical value on their desired body weight. In analyzing these data, we found that the difference between a subject's desired body weight and his or her actual body weight (a measure that captures the psychological dimensions of obesity) is a much more powerful predictor of morbidity than is BMI (a measure that captures the physiological dimensions of obesity).

Third, to be reasonably certain that stigma-induced psychological stress plays a significant role in BMI-associated mortality, we would expect to see overlap in the causes of death due to obesity and stress. As it turns out, the conditions associated with the stress response – hypertension, heart disease, type II diabetes, and hypercholesterolemia – are the very conditions associated with obesity.[6,16]

Finally, if stigma plays a major role in the association between obesity and health, we would expect less stigmatized groups to have less obesity-associated morbidity. As it turns out, younger persons, whites, and women are disproportionately affected by negative body image concerns arising from sub-group norms, and these same groups disproportionately suffer from BMI-associated morbidity and mortality.[2,21,39,40]

In short, I test my hypothesis that the stress produced by obesity-related stigma plays a major role in the pathophysiology of obesity using four very different conceptual approaches. This framework provides the reader with a gestalt of the interlocking evidence supporting the obesity-related stigma hypothesis. In the following sections, I will focus on each line of evidence in detail. I will briefly discuss less-developed forms of evidence requiring further development. I will then conclude with a discussion of the limitations of the analysis, and evidence countering the proposed hypothesis.

## Results

### The endocrinology of stress and obesity

#### Adipose tissue as an endocrine organ

Obese persons have higher serum levels of molecular compounds that promote blood clotting and inflammation.[7,10,11,13,35] This is true even after controlling for fat consumption, age, gender, race, smoking, income and medication use.[35] When serum levels of such compounds (e.g., cytokines) remain elevated for extended periods, permanent physiological changes can occur that disrupt homeostatic function.[6,16] These compounds are thought to underlie much of the pathophysiology of obesity.

Visceral fat secretes at least eleven such compounds. [7-11] However, visceral fat is composed of different types of tissue and there is considerable debate surrounding whether adipose cells themselves secrete these compounds.[8,12,13] While adipose cells share a common lineage with immune cells, they appear to lack the vesicles needed to actually store and secrete endocrine or paracrine compounds.[41] It is thought instead that these compounds are released by immune cells, which tend to surround or invade fatty tissue.[12,13] Because immune cells are both activated by the autonomic stress response and primarily responsible for releasing these compounds into the serum, it is conceivable that adipose cells play less of a role in the pathophysiology of obesity than was previously thought.

#### The neuroendocrinology of stress

Before exploring hypothetical reasons why fatty tissue might secrete pro-inflammatory, pro-thrombotic compounds, it is important to consider the function of these compounds in evolutionary biology.[16] Foremost, these compounds enhance blood clotting when the body is injured. They also provide feedback to the autonomic nervous system, leading to an increase in blood pressure and heart rate to ensure that the body can escape or fight a predator.[16,42] Finally, they mobilize the immune system to fight off infectious agents that may enter through broken skin.[16]

The endocrine molecules responsible for this pro-inflammatatory, pro-thrombotic response can take critical seconds to fully enter the circulation and reach the area of injury. Anticipation of threat by the brain therefore plays a critical role in preparing the body well in advance of any attack.[22,43,44] For instance, when an animal sees a predator, the visual information activates emotion and stress circuits in the brain.[42] These circuits then activate other neural and endocrine centers that lead to activation of the fight or flight autonomic nervous system and the release of hormones, such as cortisol, and cytokines, such as CRP. Today, most of these threats take the form of aggressive drivers, controlling bosses, annoying neighbors or domestic conflict rather than a threat of bodily harm by a predator.[45,46]

Visceral obesity might therefore increase serological levels of these mediators via obesity-induced stigma or via direct secretion. Either way, the resulting biochemical changes increase one's risk for hypertension, glucose dysregulation, and dyslipidemia.[7-9,16,47] These changes, in turn, explain the higher rate of diabetes, heart disease, hypertension, and infectious disease.

#### Competing hypotheses

There are at least three explanations for the association between adiposity and higher serum levels of molecular compounds that regulate inflammation and thrombosis.

• First, it has been proposed that these physiological changes in obese persons might occur as a side effect of the linkage between the immune system and satiety. Immune system maintenance consumes 15% of the body's daily energy needs.[48] When injured, stressed, or starved, the body must divert energy to tissue repair and shut down all functions that are not critical for survival. When well fed, levels may naturally rise.

• Second, it is possible that high stress individuals become obese as a result of excess cortisol levels and stress-induced overeating.[49] If this is the case, then stress causes obesity, rather than the other way around.

• Finally, it is possible that the psychological stress associated with the social stigma of being overweight or obese leads to a physiological stress response.[2,38]

It is likely that all three hypotheses are correct. For instance, higher serum cortisol is known to increase central obesity.[49,50] However, because weight loss appears to reduce the circulating levels of pro-inflammatory mediators, this cannot be the sole explanation for higher serum levels of mediators in obese persons.[17] Likewise, there is good evidence that rats, which are presumably free of obesity-related social stigma, produce higher levels of these compounds when overfed to the point that they become obese. Finally, obese persons experience discrimination, and stigma-induced psychological stress is a well-described phenomenon.[16,35,51] It would thus be surprising if stigma were not a contributor to obesity-associated morbidity.

#### Evidence from the stress literature

The stress literature has not yet focused on obesity. However, BMI is often included as a covariate in regression analyses examining the association between stigma and stress or socioeconomic status and stress.[28,35,36,52] This literature contributes to the debate because subjects are often tested for stress reactivity, a measure of allostatic load. Allostasis, or a shifting of the body's endocrinologic thermostat, likely happens in the brain rather than fatty tissue. Therefore, evidence of stress reactivity among obese persons would be suggestive of a link between stigma-induced stress and obesity-associated morbidity.

One common approach to measuring the stress response in the stress literature is to administer a stressful task, and then measure changes in the autonomic response, serological levels of cytokines, or changes in cortisol response (via any step in the hypothalamic-pituitary-adrenal axis).[28,52] In these studies, blood samples are collected at baseline and post-task. Where measured, BMI is usually among the most important predictors of significantly greater autonomic activation and cytokine production. This suggests that allostatic load may play a role, and may lend evidence to the hypothesis that some obesity-associated illness arises from perceived stigma. If so, it is useful to examine how variations in perceived stigma might explain variations in physiological responses among obese persons.

### Body preferences and the distribution of disease

Body norms differ by gender, race, ethnicity, age, and socioeconomic status.[38-40,53,54] For instance, heavier women are more likely to have a negative body image than heavier men. Likewise, white race, young age, high income, and high educational attainment are all predictors of body dissatisfaction among overweight persons. Conversely, overweight African-Americans, Hispanics, young persons and men all tend to be more comfortable with their bodies when they are overweight or obese.

If perceived stigma is responsible for some of the obesity-associated morbidity and mortality, differences in body size preferences by social class, age, race, and gender should translate into differences in morbidity and mortality. In the following sections, I will review patterns of excess morbidity and mortality among overweight and obese persons by gender, race, and age.

#### Gender and weight-related health

Women are seven times as likely as men to experience excess quality-adjusted life years (QALYs) lost to being overweight.[2] (A QALY is a measure that combines morbidity and mortality, with one QALY equal to one year of perfect health.) These effects are seen both for broad measures of health, as well as for specific diseases, such as depression.[21,55]

In a comprehensive burden of disease analysis by gender, I collaborated with a research team to analyze the Medical Expenditure Panel Survey (MEPS).[2] The objective was to ascertain both the morbidity and mortality effects of being overweight (BMI > 25 to ≤ 30) or obese (BMI > 30) by gender. The MEPS is a large, nationally representative survey that contains a health-related quality of life instrument, which can be used to estimate QALYs. This instrument, the EuroQol, measures "health" with respect to mobility, self-care, activity limitations, anxiety/depression, and pain/discomfort. Thus, it captures the extent to which people are anxious or depressed about their weight as well as any impacts obesity might have on other aspects of their physical health.

**Figure 1:**
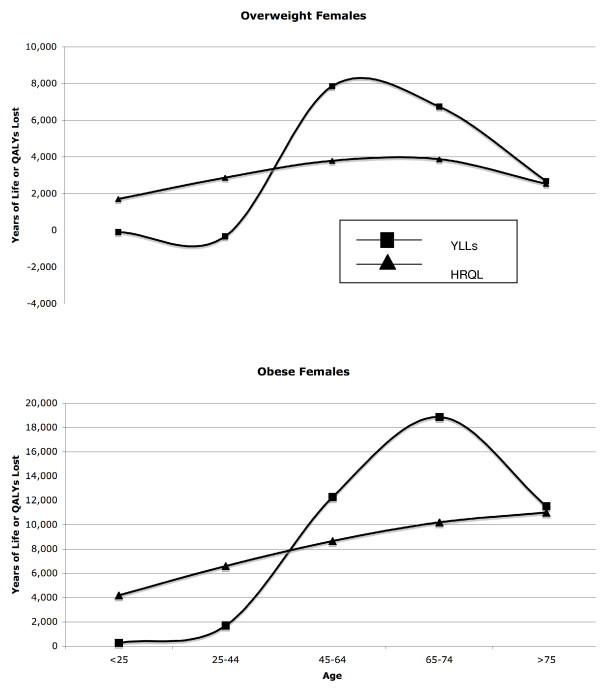
**Relationship between excess years of life lost to mortality (YLLs) and excess morbidity (HRQL) among overweight (BMI 25–30) and obese (BMI 30+) women by age.** In both weight categories, women suffer cumulative increases in excess morbidity (both psychological and physiological) until they reach their 30s, at which point, excess mortality increases.

We found that younger overweight and obese women tend to be much less healthy than normal weight women up to around age 45. However, despite their poorer health, heavier young women experience similar or even lower numbers of deaths as normal weight women in this age group. Much of this excess morbidity among younger overweight women may be psychological, taking the form of anxiety and depression.[55] After age 45, excess morbidity declines and excess deaths begin to rise (Figure 1). This pattern roughly fits with a stress-based model of disease; both race-associated stigma and the stressors associated with low socioeconomic status show a similar pattern of excess morbidity and mortality. [56-58]

#### Race and weight-related health

**Figure 2:**
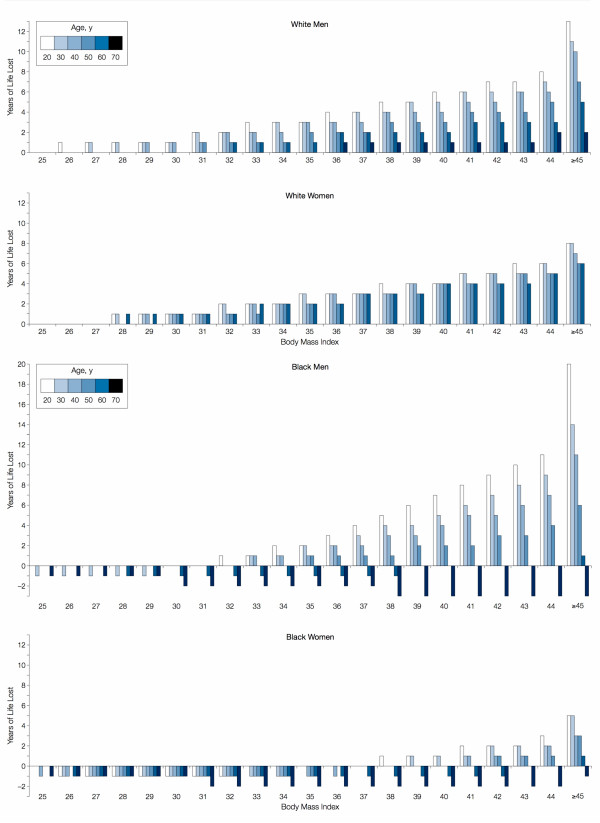
**Life years lost by body mass index, age, and race.** (Figure from Fontaine et al, 2003, used with permission.).

As might be predicted by the distribution of negative body image, whites suffer from excess mortality at a much lower BMI than African-Americans.[21] In Figure 2, we see that white men and women begin to experience excess loss of life at a BMI of typical of overweight persons (BMI > 25 to ≤ 30). For African-Americans, being overweight is protective among both men and women. Moreover, obesity (BMI ≥ 30+) is associated with excess years of life lost only among younger African-Americans.

In fact, African-American women who have a BMI of 30 to 37 suffer fewer excess years of life lost than normal weight African-American women across all age groups. Moreover, African-American women do not experience excess mortality unless they are less than 60 years of age *and *over a BMI of 37. If adiposity is a health risk in this group, there must be a very powerful factor driving survival among overweight African-American women. This is unlikely to be socioeconomic status, given that low-income African-American women are more likely to be overweight than high-income African-American women.[56]

#### Age and weight-related health

There has been a long-term trend toward negative body image among heavier females.[59] Fuller-figured models were still preferred in the World War II era, with thin bodies possibly signifying inadequate nutrition.[60] However, as the economy gained distance from the depression era, thin bodies may have come to signify membership in the leisure class. World War II generation women appear to be somewhat spared from these effects; women in the 65 and over age range report less negative body image and experience less excess morbidity or mortality than younger women.[21]

It is possible, however, that this represents little more than a generic survival effect. If so, we would expect to see less survival effect by age among older African-American females (since this group experiences less mortality than whites at younger ages). However, even morbidly obese elderly African-American women have fewer excess years of life lost than their "normal" weight peers (Figure 2).

Mortality data by gender, race, and age strongly support the hypothesis that sub-group norms surrounding body image have a strong influence on premature mortality. However, they also raise some questions. For one, we do not know how socioeconomic status influenced the observed rates. While poor body image is positively associated with income and educational attainment, BMI is negatively correlated with these variables.[2] This would suggest that the observed effects are stronger than the graphs show, but it also demonstrates the complexity of such an analysis, and the potential for confounding. We would want to see more direct evidence that discord between one's desired weight and one's actual weight is a predictor of morbidity and mortality.

### BMI versus desired weight

Consider that it is weight dissatisfaction that is driving the relationship between BMI and health, and that BMI is a spurious confounder. If so, then it should be possible to design a study that measures subjects' weight satisfaction, BMI, and health status.[38] One could then ascertain the relative contribution of BMI and body weight satisfaction to health status to ascertain which exerts more of an effect.

The 2003 Behavioral Risk Factor Surveillance System (BRFSS) provides the opportunity to conduct just such an analysis. This dataset is based on a telephone survey of over 247,000 persons nationwide. Key outcome measures in the BRFSS include the number of physical or mental "unhealthy days" experienced in the past month. The 2003 BRFSS contains information both on subjects' reported BMI and their desired body weight. The latter is measured with the question, "How much would you like to weigh?" The difference between the desired weight and actual weight is a relatively straightforward measure of one's desire for a thinner body.

In this study, all subjects who were below a BMI of 23 (approximately the mid-point of the "normal" range) were removed to ensure that no anorexic subjects were included.[38] Separate models were built by gender and race.

The findings in this analysis were also consistent with the obesity-related stigma hypothesis; the desire to lose weight is a much stronger predictor of morbidity than actual BMI in all models. Moreover, the relationship is stronger for women than men, and for whites than African-Americans or Hispanics. Again, this is consistent with data from body image surveys.[40]

Controlling for BMI and age, males who wish to lose 1%, 10% and 20% of their body weight suffer a net increase of 0.1, 0.9, and 2.7 total unhealthy days per month. For females, the corresponding numbers are 0.1, 1.6, and 4.3 total unhealthy days per month. In some models, a higher BMI is predictive of *fewer *unhealthy days than a lower BMI once the relative desire to lose weight is controlled for.

This finding potentially explains the survival advantage seen among some overweight or moderately obese groups in the medical literature.[21] If some groups see being overweight in a positive way, it may confer a survival advantage. It has also been hypothesized that overweight persons are more likely to be screened for diabetes and hypercholesterolemia than normal weight persons and that this explains the improved survival.[61] However, this would not explain why overweight African-Americans are at a significantly greater survival advantage than overweight whites, since whites have better access to medical care. It is also simply possible that overweight people get a better cardiovascular workout doing day-to-day activities than normal weight persons. In short, being moderately overweight may be healthy, but it is those groups that tend not to see fat as unattractive realize health benefits.

If a distorted body image leads to stress and thus morbidity, we would expect that young males who see themselves as too skinny would also suffer.[62] In post-publication analyses, Rufina Lee and Marilyn Sinkowitz of Columbia University, explored whether the desire to gain weight also predicted greater physical and mental unhealthy days among young males. Here, too, a measure of distorted body image – in this case, the desire to *gain *weight – was correlated with psychological and physiological morbidity among males aged 18 to 30 (unpublished results).

These data provide compelling support to the hypothesis that stigma plays a contributing role in the health problems associated with the obesity epidemic. However, it is also possible that society's heightened awareness of the health risks associated with obesity explain the effects observed in this study. For instance, some subjects may wish to lose weight simply because they were previously diagnosed with hypertension, heart disease, or some other condition for which weight is thought to be a risk factor. If so, the observed association between the desire to lose weight and physical unhealthy days could be due to pre-existing conditions.

### Other lines of evidence

In this section, I will briefly describe some other areas of research that show promise, but require further exploration. The first is the case of Mauritania. Mauritania so values heavy women that girls are often force-fed at a young age and are often administered steroids, such as prednisone, to gain weight. This country is currently in the lowest prevalence category for diabetes.[63] However, very little is known about the distribution of diabetes within the population. Most importantly, it would be helpful to have data on subjects that became overweight without the help of glucocorticoids, which can alter the risk of diabetes. It would also be helpful to have data on temporal trends. Specifically, it would be useful to observe whether, as the country is increasingly exposed to North American and European cultural norms, fat is stigmatized. If so, we may expect acculturation from the west to lead to a diabetes epidemic in this country. It would be equally useful to compare "metrosexuals," straight urban males who tend to be concerned about their appearance,[64] to other males.

The second area that requires further exploration is the relationship between outward appearance and actual body fat. We do know that visceral body fat predicts pathophysiology, but that subcutaneous body fat does not.[9,65] It is plausible that visceral body fat causes more body shape distortion, and therefore more concern over the changes in one's body.

Likewise, persons who are "big boned," or broad in form can appear fat even when they are actually lean. Such persons will also have an artificially elevated BMI. Thus, BMI may be a better predictor of whether people appear fat than it is of their actual total body fat.

Bioelectrical impedance analysis (BIA) is a highly accurate measure of total body fat that is not readily apparent to the naked eye. If adiposity explains the relationship between BMI and health, we would expect BIA to be a better predictor of obesity-associated conditions than BMI. Here, too, it appears as if BMI is a stronger predictor of obesity-associated conditions than BIA.[66] Thus, if body shape and size were more important than actual body fat in determining pathophysiology among obese persons, we would expect that stigma-induced stress is again a potential culprit. However, more detailed analyses are needed. For instance, it would be helpful to control for variance in body fat distribution by age, race, and gender and to have a more concrete measure of perceived body image in BIA studies.

## Conclusion

It is important to understand the role that stigma plays in producing disease amongst overweight and obese persons for many reasons. Foremost, the assumptions surrounding the pathophysiology of overweight and obesity could be partially incorrect. Second, if stigma-induced stress plays a role in the pathophysiology of obesity, it suggests that social constructs of idealized body image can have harmful health effects. If so, it raises pragmatic questions surrounding the net health benefits of public health communications campaigns, which often promote thinness. Finally, overweight and obesity are associated with over 7 million QALYs annually in the U.S., potentially making it one of the major causes of death.[2]

Nonetheless, the evidence I appraise here is far from conclusive. First, there is evidence that fatty tissue does have metabolic function. This is true not only of steroid production and function,[47] but may also be true of cytokine production. [4-13] Further work in this area will help clarify the relationship between immune tissue and fatty tissue in the production of pro-inflammatory and pro-thrombotic mediators.

Complementary hypotheses also exist. For instance, there is strong evidence that stress increases the risk of central obesity.[49,50] Thus, there is not only room for confounding in some of the data I discuss, but the findings are partially explained by reverse causality. As discussed above, reverse causality can only play a limited role, since dieting can reverse the metabolic effects of obesity.[17] However, neither hypothesis explains why a discrepancy between one's perceived ideal weight and his or her actual weight is a stronger predictor of morbidity than BMI.[38] Nor do they explain why differences in body image norms among different racial and ethnic groups appear to be strong predictors of obesity-related mortality.

There are other reasons why the obesity-induced stigma hypothesis cannot explain all of the association between obesity and morbidity. For instance, there is evidence that adiposity is independently linked to some obesity-related illnesses.[67] In particular, there is a link between internal obesity, which is less visible, and the metabolic syndrome in Asians.[68] This suggests that genes, developmental environment, or reverse causality play a role in this group. Moreover, genetic makeup in general might simultaneously affect one's propensity to gain weight as well as his or her physiologic response to being overweight.[69] Nevertheless, the obesity-related stigma hypothesis is logical and complements, rather than refutes, the adiposity hypothesis.

There are certainly conditions and illnesses that can be clearly attributed to obesity. One of these is sleep apnea, which has also been linked to metabolic disruptions.[70] Sleep deprivation has also been linked to excess stress mediator production in high stress populations, such as persons of low socioeconomic status. This highlights the many psychophysiological pathways at work in producing obesity-associated morbidity.

One final concern is that a wide array of other complex psychosocial factors contributes to obesity-associated morbidity and mortality, and it is difficult to tease them apart. For instance, stress can reduce cognitive function via damage to the hippocampus (a region of the brain important in memory), potentially contributing to heavy persons' lower educational attainment.[71,72] Lower educational attainment, in turn, may explain some of the health and longevity effects of obesity. Likewise, stress can play a role in obese persons' higher incidence of smoking. Like stress, smoking is associated with dyslipidemia, atherosclerosis, and elevated CRP levels.

In developing nations where food is scarce, obesity might be seen as a proxy measure for affluence (and thus dominance in social hierarchies). As nations industrialize, however, cheap, calorie-dense food becomes plentiful and leisure time becomes scarce. Thus, thinness replaces plumpness as a proxy measure for affluence. By looking at case studies such as Mauritania, which still values plumpness, we should be able to observe an increase in obesity-associated pathophysiology that is coincident with shifts in depictions of beauty in the media.

If shifts in depictions of beauty in the media are partially to blame for the health effects of obesity, public health officials may have unwittingly exacerbated the problem by promoting thinness. This paper's hypothesis thus speaks to larger examinations of public health policy, and health communications campaigns. If the obesity epidemic is partially attributable to social constructs surrounding ideal body types, there is a need for new research and policy paradigms that emphasize fitness and healthy eating habits[73] alongside social acceptance of heavier members of society.

## Pre-publication history

The pre-publication history for this paper can be accessed here:


